# The Emerging Role of Osteopathic Manipulative Medicine in Enhancing Quality of Life for Palliative and End-of-Life Patients: A Narrative Review

**DOI:** 10.7759/cureus.93306

**Published:** 2025-09-26

**Authors:** Ambrose Loc T Ngo, Linda Nguyen, Cynthia Shahbandeh, Jared Nichols

**Affiliations:** 1 College of Osteopathic Medicine, Kansas City University, Joplin, USA; 2 College of Pharmacy, Western University of Health Sciences, Pomona, USA; 3 Osteopathic Manipulative Medicine, Kansas City University, Joplin, USA

**Keywords:** acute pain management, end of life and hospice care, osteopathic care, osteopathic manipulative medicine, supportive and palliative care

## Abstract

Palliative and end-of-life care aim to minimize suffering and optimize the quality of life for individuals nearing the end of their lives. Osteopathic manipulative medicine (OMM) represents a potentially valuable nonpharmacological approach within this integrative care framework. Emerging evidence from clinical observations, small-scale studies, and foundational osteopathic principles suggests that OMM may help support patient comfort, alleviate certain symptoms, and contribute to dignity in care. Techniques such as myofascial release, cranial manipulation, and lymphatic drainage have been reported to improve comfort and emotional well-being; however, these findings are based primarily on limited and heterogeneous studies. This narrative review synthesizes current clinical evidence on the application of OMM in palliative and end-of-life care, focusing on its potential to manage multiple distressing symptoms, including pain, respiratory distress, fatigue, gastrointestinal issues, edema, and psychological stress. The review aims to provide an integrative understanding of OMM’s role in symptom management, identify evidence gaps, and propose directions for future research.

## Introduction and background

Care for chronic illnesses employs various approaches to relieve patient discomfort. Palliative care specifically focuses on easing suffering and enhancing quality of life for patients with serious illness, while end-of-life care is directed toward those nearing death. Both disciplines emphasize symptom management, psychosocial support, and overall wellness [1,2]. The concept of "total suffering," introduced by Cicely Saunders, captures the multidimensional nature of patient distress, encompassing physical, emotional, social, and spiritual challenges [3]. Holistic and interdisciplinary approaches that address multiple symptom domains have been shown to improve patient-reported outcomes.

Osteopathic manipulative medicine (OMM), a core component of osteopathic practice, emphasizes the interconnectedness of body structure and function, aligning with the principles of addressing the whole person [4,5]. Although initially focused on musculoskeletal conditions, OMM has increasingly been applied to chronic disease management and rehabilitation. Given the overlap between chronic illness and palliative care populations, these developments support the growing interest in exploring OMM within palliative contexts [6]. This narrative review, therefore, aims to comprehensively synthesize existing evidence on the application of OMM in palliative and end-of-life care, with a specific focus on its role in managing key symptoms such as pain, respiratory difficulties, gastrointestinal distress, and emotional well-being. By addressing these multiple symptom domains, the review seeks to provide an integrative perspective on how OMM may contribute to improving patient comfort and quality of life. This approach acknowledges the multidimensional nature of suffering in palliative care and positions OMM as a potentially valuable adjunct in symptom management within this complex context.

## Review

Methods

Study Protocol and Sources

In developing this narrative review, we sought to explore how OMM has been applied within palliative care, with particular attention to its role in symptom management and quality of life. The literature search was conducted primarily using PubMed and Google Scholar databases, chosen for their accessibility and broad coverage of biomedical and osteopathic research. While we recognize that other databases such as Embase, Cochrane Library, CINAHL, and PsycINFO may provide additional relevant studies, the scope of this narrative review was constrained to these two platforms to focus the search and maintain feasibility. A detailed search strategy, including key terms related to OMM, palliative care, and end-of-life care, was employed. Boolean operators (AND, OR) were used to combine terms such as "osteopathic manipulative treatment," "palliative care," "end-of-life," "pain management," and "symptom relief." Searches were limited to articles published in English within the last two decades to capture the most clinically relevant and recent data.

Articles retrieved were screened by title and abstract for relevance, followed by full-text review to identify studies focusing on the integration of OMM into palliative care. We supplemented database searches with a manual review of references to identify additional pertinent studies. Although a formal PRISMA (Preferred Reporting Items for Systematic Reviews and Meta-Analyses) flowchart was not generated due to the narrative nature of this review, we documented study selection steps to ensure reproducibility.

Risk of Bias Assessment

For included clinical trials, the risk of bias was assessed using appropriate tools such as the Cochrane Risk of Bias 2.0 (RoB 2.0) for randomized controlled trials (RCTs) and ROBINS-I (Risk of Bias in Non-randomized Studies of Interventions) for non-randomized studies. Consistent with RoB 2.0 terminology, studies were categorized as having “low risk,” “some concerns,” or “high risk” of bias rather than the previously used term “moderate.” These assessments are summarized in Tables 1, 2, highlighting potential limitations affecting the interpretation of results.

Inclusion and Exclusion Criteria

The scope of this review was limited to the last two decades, although more recent studies were preferred. All articles had to be in the English language and pertain to the field of palliative care, focusing on patients who were subjected to OMM. RCTs, cohort studies, case-control studies, systematic reviews, books, and meta-analyses on the impact of OMM on symptom management, motor function, pain alleviation, mobility, and quality of life were accepted. Excluded articles included those focusing on non-OMM manipulative therapies (such as chiropractic or generic physical therapy), editorials, opinions, preprints, and unpublished documents. Moreover, non-OMM manipulative therapies were not included in this analysis. 

Study Selection

Our search of the literature yielded a broad collection of studies from both PubMed and Google Scholar. After removing duplicates and excluding works that did not meet the inclusion criteria, we focused on those most relevant to the scope of this review. Through careful screening of titles, abstracts, and full texts, a final set of articles was identified and incorporated into the synthesis. Additionally, we considered the overall quality of the evidence by assessing potential sources of bias. For studies that included clinical trials, standardized tools such as the Cochrane risk of bias and ROBIN-I frameworks were applied to evaluate methodological rigor and highlight limitations that may influence interpretation, as shown in Tables 1, 2. This allowed the review not only to synthesize reported outcomes but also to contextualize the strength and reliability of the evidence base.

**Table 1:** Cochrane ROB bias analysis on randomized controlled trials. ROB: Risk of Bias.

Study	Randomization Process	Deviations From Intended Interventions	Missing Outcome Data	Measurement of the Outcome	Selection of Reported Result	Overall Risk of Bias
Galeazzi et al. 2025 (*Pain Rep*, palliative OMT vs sham) [6]	Low	Low	Low	Low	Some concerns	Some concerns
Nguyen et al. 2021 (*JAMA Intern Med*, LBP OMT vs sham) [7]	Low	Low	Some concerns	Low	Low	Some concerns
El-Khateeb et al. 2022 (*Eur J Phys Rehabil Med*, neck pain SCS add-on) [8]	Low	High	Some concerns	High	Some concerns	High risk

**Table 2:** ROBIN-I bias analysis on non-random trials ROBIN-I: Risk of Bias in Non-randomized Studies of Interventions.

Study	Confounding	Selection of Participants	Classification of Interventions	Deviations From Intended Interventions	Missing Data	Measurement of Outcomes	Selection of Reported Result	Overall Risk of Bias
Arienti et al. 2018 (*Integr Cancer Ther*; nonrandomized oncology geriatrics trial) [9]	High risk	Some concerns	Low	Some concerns	Low-some concerns	Serious	Low-some concerns	High risk

Results

End-of-Life and Palliative Care: Clinical Needs and Challenges 

Palliative care’s approach encompasses symptom relief, compassion, spiritual care, and teamwork [9]. It strives to preemptively relieve suffering by managing pain and other distressing symptoms, assessing them, and treating them [10]. There is a complex symptomatology at the end of life, arising from various underlying pathologies, which includes pain, severe fatigue, immobility, dyspnea, edema, gastrointestinal discomfort, and other profoundly distressing symptoms [11]. These symptoms may be caused by disease progression, aggressive treatment, and chronic inability to perform daily activities. Additionally, addressing these demanding symptoms through a singular lens, such as medicines, is a growing unaddressed concern. Although medication is a crucial aspect, it comes with a range of adverse effects such as sedation, constipation, and cognitive impairment, which may worsen a patient’s quality of life [12]. Given this, physicians must navigate ethical considerations carefully, balancing beneficence, defined as "actively relieving patient suffering," and non-maleficence, defined as "avoiding harm from overly aggressive interventions." Compared with pharmacological treatments that carry risks of sedation and cognitive impairment, OMM is considered less aggressive due to the gentle, non-invasive nature of the possible techniques.

OMM: Principles, Techniques, and Application in Palliative Care

OMM is rooted in osteopathic philosophy. It focuses on the interrelation of the body's systems, self-regulatory capabilities, and the holistic care framework of an individual, rather than a disease-specific approach to treatment [4]. This ideology is congruent with palliative care, which similarly prioritizes holistic, patient-centered strategies rather than solely disease-focused interventions, aiming to address comprehensive patient well-being. Most published studies in this field are of low to moderate quality (case series and case reports), with only one RCT identified. In palliative settings, OMM offers both patience and flexibility, referring to its adaptability in intensity, duration, and technique selection, which allows physicians to safely address the unique physical limitations and varying symptom severities commonly seen in fragile palliative patients. Common techniques include soft tissue treatment, myofascial release, lymphatic drainage, counterstrain, and osteopathic cranial manipulative medicine (OCMM) [4,5]. These actions aim to reduce tissue strain, increase blood and lymph circulation, balance autonomic nervous system activity, and induce relaxation. When applied in end-of-life care, OMM may help manage skeletal and musculoskeletal pain, respiratory obstruction, fluid retention, and stress-related bodily symptoms [13]. Additionally, OMM is unique in that it provides patients with a reparative encounter that fosters connection, trust, and compassion, which are crucial for a person reaching the end of their life.

Pain Management With OMM in Palliative Care

Pain is one of the most distressing and prevalent symptoms experienced in terminal illness. It may arise from bone metastases, nerve involvement, visceral compression, or musculoskeletal dysfunction. The primary non-invasive choice for pain relief in palliative settings remains opioids, though their use carries risks such as sedation, constipation, nausea, altered cognition, and potential for addiction or tolerance [14,15]. These adverse effects have fueled interest in non-pharmacologic interventions, including physical therapy, massage, acupuncture, and mindfulness practices. However, the accessibility, consistency, and effectiveness of these options are limited, leaving a potential role for OMM as an adjunctive therapy. OMM offers a range of non-invasive techniques tailored to individual patient needs. Soft tissue and myofascial release techniques relax muscular tension and fascial restrictions, increasing range of motion and mobility [16,17]. Counterstrain methods address tender points caused by muscle spasms and strain, while cranial techniques aim to alleviate tension headaches and central nervous system irritability [7,18,19]. Collectively, these interventions may help reduce somatic dysfunction and could complement pharmacologic pain management, although further controlled studies are needed.

Galeazzi et al. performed an RCT that evaluated the efficacy of OMM versus sham therapy in 75 patients with advanced-stage cancer in a palliative care unit [6]. After three withdrawals, 37 patients in the OMM group and 38 in the sham group were analyzed. Treatments were administered every 2 days for 7 days, with pain measured via the Visual Analog Scale (VAS) and quality of life assessed using QLQ-C15-PAL. OMM produced a statistically significant reduction in VAS scores, starting on day 3 (D3 pm, P = 0.03) and continuing through day 6 (D6 pm, P = 1.28 × 10⁻⁵), corresponding to a 43.2% overall improvement. Among patients using controlled analgesia pumps, analgesic demand decreased by 31.6% (P = 0.016) [6]. Quality-of-life scores trended toward improvement by day 6, and no adverse effects were reported. These results provide the strongest evidence to date supporting OMM as an adjunctive pain management strategy in palliative care. Lower-level evidence from case series and observational studies corroborates these findings, reporting improvements in patient comfort and reduced reliance on breakthrough medications [20]. Together, the data suggest that OMM can meaningfully reduce pain, decrease opioid use, and provide a supportive, humanistic component to end-of-life care.

Respiratory and Lymphatic Support Through OMM

End-of-life care is recognized to be associated with significant respiratory symptoms such as dyspnea and shallow breathing, which contribute greatly to patient distress [21]. Additionally, cancer patients often suffer from pulmonary complications due to metastasis, infection, fluid buildup, or immobility [22,23]. Lymphatic drainage is particularly important because it helps clear excess fluid buildup from tissues, reducing uncomfortable swelling and congestion. By increasing lymphatic flow, breathing difficulty can be reduced, helping patients feel more comfortable and less anxious. OMM can help improve thoracic mechanics and lymphatic drainage, increasing ease of respiratory effort and oxygenation. Diaphragm movement, rib raising, thoracic and pedal lymphatic pumps, diaphragm release, and thoracic inlet release can also increase thoracic compliance and clear fluid from congested lungs [24,25]. Even patients with advanced disease and very limited mobility due to poor musculoskeletal strength have been shown to subjectively and objectively improve their respiratory function through the application of these techniques [25,26]. The gentleness of these applied techniques makes them ideal for the most fragile patients.

OMM for Fatigue, Gastrointestinal Symptoms, and Edema

Along with pain and dyspnea, terminally ill patients frequently endure fatigue, constipation, bloating, and edema, which profoundly impact quality of life. Fatigue in palliative care may be multifactorial due to altered metabolism, inadequate dietary intake, immobility, and emotional burden [27]. OMM offers a range of non-invasive techniques that may physiologically support these systems and complement standard care. Gentle myofascial and soft tissue techniques may restore circulation and alleviate musculoskeletal strain that contributes to fatigue [4]. Requisite to achieving parasympathetic activity is visceral manipulation and abdominal lymphatic drainage, which may enhance motility and relieve constipation and bloating, without the use of additional pharmaceuticals [4,28]. Recent clinical observations suggest that patients receiving OMM may experience improvements in energy levels, reduced swelling, and relief from gastrointestinal discomfort [4,28]; these findings remain preliminary and hypothesis-generating. One randomized study in a non-palliative cohort (n = 52; 16 males, 36 females) comparing manual lymph drainage (MLD), abdominal massage (AM), and electrical stimulation (ES) demonstrated that MLD significantly improved bowel movement frequency (p < 0.1), bowel movement duration (p ≤ 0.02), and autonomic nervous system measures including total power and high-frequency HRV (p ≤ 0.01) [28]. While this study was not conducted in a palliative population, its findings support the potential of lymphatic manipulation techniques to relieve constipation and modulate autonomic balance. These interventions may enhance functional status, promote a sense of lightness and mobility, and provide non-pharmacologic symptom relief. While larger trials are warranted, currently available evidence suggests OMM may more comprehensively address these unrecognized symptoms, often overshadowed by more acute pain or respiratory distress.

Emotional and Psychological Benefits of OMM

At the end of life, psychological distress is a major obstacle that every patient is forced to endure. It requires a whole range of emotions, including anxiety, depression, fear, and emotional tension, which are physically expressed and can worsen pain and fatigue [29-31]. OMM targets both the physiological and emotional suffering derived from the stressors of chronic disease by balancing the autonomic nervous system through osteopathic techniques while also fostering human connection through direct physician-patient interaction. Soft tissue as well as cranial manipulation can trigger the parasympathetic nervous system, calm sympathetic overactivity, and usher in a calming restorative state [32,33]. Moreover, OMM provides affirmation and reassurance, which may contribute to enhanced relaxation and reduced anxiety; however, it is unclear how much of these effects are attributable to specific osteopathic techniques versus general therapeutic touch or attention [32].

Enhancing Quality of Life and Preserving Dignity

The primary aim of palliative care is to maintain or improve the quality of life of the patient [8], and this includes preserving the dignity of patients. Dignity in a healthcare setting can be defined as a patient's sense of self-worth and autonomy, often negatively affected by dependency, physical limitations, or emotional instability. Physicians support patients in regaining dignity by providing treatments and interactions that acknowledge their individual needs, respect their autonomy, and emphasize their worth. OMM can help enhance functional capacity as well as ease discomfort, enabling a calmer and more comfortable dying process by addressing various physical and emotional aspects [4]. By improving comfort and alleviating distressing physical symptoms, OMM can enable patients to engage more meaningfully in interactions with family members and loved ones, significantly enhancing their quality of life during their final days. OMM can also help maintain patients' mobility by reducing stiffness and muscular discomfort. By enhancing mobility, patients gain the freedom to participate more actively in meaningful everyday activities such as sitting up to talk with visitors, engaging in hobbies, or simply maintaining basic self-care, which significantly prolongs their independence and sense of autonomy near the end of life [4]. The utilization of manipulative techniques can promote comfort and dignity to a bedbound patient or one in the end-of-life process. Even when curative treatment is no longer an option, OMM can serve to convey dignified and compassionate care to the patient. Additionally, there is some emerging literature and case observation that support OMM as a useful intervention to enhance patient satisfaction and perceived quality of care [34,17].

OMM in Multidisciplinary Palliative Care

The addition of OMM to palliative care teams broadens the range of services that patients may receive. Because OMM treatments avoid medications or invasive procedures, they are gentle and low risk, enhancing their suitability for frail populations and those with complex comorbidities and multifaceted chronic illnesses [4,35]. The inclusion of osteopathic physicians in hospice and palliative teams cultivates a more integrative approach toward patient care. OMM can be utilized alongside palliative therapies like analgesic medication, radiation therapy, and psychological support [6,36]. When applied by osteopathic physicians, it is in keeping with the interdisciplinary philosophy of palliative care that invites multidisciplinary collaboration with nurses, social workers, chaplains, and rehabilitation specialists [4,8,36]. Such incorporation fosters holistic care that addresses the numerous requirements of patients and families. Educating and exposing both healthcare professionals and the public to OMM would normalize its use in standard palliative and hospice care, allowing more patients to access its benefits [8,37,38]. While integrating OMM into palliative care teams appears promising, practical considerations remain. These include physician availability, training requirements, treatment time allocation, and reimbursement logistics. Economic analyses and workflow assessments are necessary to evaluate the cost-effectiveness and feasibility of widespread OMM implementation in multidisciplinary palliative care settings. Incorporating the OMM systemically would not only recognize these techniques as a valid option but also standardize training and practice, benefiting both patients and providers.

Future directions

While OMM appears to improve patient comfort and quality of life in palliative and end-of-life settings, the current evidence base remains limited. Future research should prioritize well-designed RCTs that evaluate not only patient outcomes, such as symptom relief and emotional well-being, but also family caregiver experiences, including caregiver burden and satisfaction with care. Additionally, studies should explore the role of OMM in reducing medication use, lowering hospital readmissions, and supporting the interdisciplinary care team.

Cross-cultural factors must also be examined to ensure that OMM practices are inclusive and applicable across diverse populations and care models. Standardization of treatment protocols, including session frequency, duration, and specific techniques, is essential for reproducibility and clinical translation. Evaluating the cost-effectiveness of OMM compared to conventional care could also inform its potential integration into healthcare systems. Finally, rather than recommending widespread adoption at this stage, targeted educational efforts and the development of clinical guidelines could lay the groundwork for future incorporation of OMM into palliative care, contingent upon stronger evidence.

Limitations 

Access to some potentially relevant articles was limited due to paywalls and database restrictions, which may have constrained the comprehensiveness of the review. Additionally, many of the included studies had small sample sizes, reducing statistical power and possibly affecting the reliability of their conclusions. The generalizability of findings is also limited by variability in patient populations, clinical settings, symptom severity, and treatment responses. While studies were grouped by subtopic to facilitate thematic synthesis, we prioritized more recent and methodologically rigorous publications. In some cases, subtopics were only included if at least three relevant articles were identified. We acknowledge that this threshold is somewhat arbitrary and may have introduced bias; future reviews with broader access and inclusion criteria may yield more comprehensive coverage. Overall, the limited number of high-quality studies on the use of OMM in palliative and end-of-life care highlights the need for further research in this area.

## Conclusions

Overall, OMM has the potential to be a beneficial method in palliative and end-of-life care. This approach’s capability to relieve pain, manage respiratory and gastrointestinal functions, mitigate fatigue and swelling, and promote wellness could support the intent of holistic, patient-centered care. Although more investigation is needed, the current body of evidence, along with numerous positive clinical outcomes, indicates that OMM is a valuable approach when considering comfort, dignity, and quality-of-life enhancement for patients in the last stage of life. Relieving suffering and protecting the dignity of individuals at the end of life is critical, and the development of palliative medicine has provided an integrative approach. With the addition of OMM, palliative medicine has significantly progressed toward a more caring and complete healthcare model for patients during this crucial, emotionally and physically challenging stage of their lives.
